# Effect of environmental enrichment and isolation on behavioral and histological indices following focal ischemia in old rats

**DOI:** 10.1007/s11357-021-00432-z

**Published:** 2021-08-12

**Authors:** Andrei Gresita, Ruscu Mihai, Dirk M. Hermann, Flavia Semida Amandei, Bogdan Capitanescu, Aurel Popa-Wagner

**Affiliations:** 1 Department of Neurology Chair of Vascular Neurology and Dementia, University of Medicine Essen, Essen, Germany; 2 Griffith University Menzies Health Institute of Queensland, Gold Coast Campus, Southport, QLD 4222 Australia; 3 Doctoral School, University of Medicine and Pharmacy, Craiova, Romania

**Keywords:** Aging, Cerebral ischemia, Enriched environment, Behavioral recuperation, Neuroinflammation, Neuroepithelial cells, Neurogenesis

## Abstract

Stroke is a disease of aging. In stroke patients, the enriched group that received stimulating physical, eating, socializing, and group activities resulted in higher activity levels including spending more time on upper limb, communal socializing, listening and iPad activities. While environmental enrichment has been shown to improve the behavioral outcome of stroke in young animals, the effect of an enriched environment on behavioral recuperation and histological markers of cellular proliferation, neuroinflammation, and neurogenesis in *old* subjects is not known. We used behavioral testing and immunohistochemistry to assess the effect of environment on post-stroke recovery of young and aged rats kept either in isolation or stimulating social, motor, and sensory environment (( +)Env). We provide evidence that post-stroke animals environmental enrichment ( +)Env had a significant positive effect on recovery on the rotating pole, the inclined plane, and the labyrinth test. Old age exerted a small but significant effect on lesion size, which was independent of the environment. Further, a smaller infarct volume positively correlated with better recovery of spatial learning based on positive reinforcement, working and reference memory of young, and to a lesser extent, old animals kept in ( +)Env. Histologically, isolation/impoverishment was associated with an increased number of proliferating inflammatory cells expressing ED1 cells in the peri-infarcted area of old but not young rats. Further, ( +)Env and young age were associated with an increased number of neuroepithelial cells expressing nestin/BrdU as well as beta III tubulin cells in the damaged brain area which correlated with an increased performance on the inclined plane and rotating pole. Finally, ( +)Env and an increased number of neurons expressing doublecortin/BrdU cells exerted a significant effect on performance for working memory and performance on the rotating pole in both age groups. A stimulating social, motor and sensory environment had a limited beneficial effect on behavioral recovery (working memory and rotating pole) after stroke in old rats by reducing neuroinflammation and increasing the number of neuronal precursors expressing doublecortin. Old age however, exerted a small but significant effect on lesion size, which was independent of the environment.

**Supplementary Information:**

The online version contains supplementary material available at 10.1007/s11357-021-00432-z.

## **I**Introduction

Age is the only one non-modifiable risk of cerebral ischemia. In recent years, due to increases in the number of the elderly, the incidence of stroke has increased again paralleled by an increase in the number of stroke survivors, many with severe disabilities, that has led to an increased economic and social burden in society. While some limited recovery is known to occur spontaneously during the first 3 months month after stroke, physical exercise and environmental enrichment stimulate task-specific neuroplasticity and spontaneous recovery [1]. In stroke patients, the enriched group that received stimulating physical, eating, socializing, and group activities resulted in higher activity levels including spending more time on upper limb, communal socializing, listening, and iPad activities [2, 3].

Experimental evidence suggests that experimental animals exposed to enriched environment-mediated motor and sensory stimulation may benefit of enhanced behavioral recovery after brain injury [4]. Likewise, transcranial magnetic stimulation in association with ( +)Env leads to functional improvement after TBI via cortical excitability and reorganization [5]. However, the mechanisms underlying the beneficial effects of the enriched environment ( +)Env in animal models are not precisely known. Thus, using laser speckle imaging, it was found that an exposure to environmental enrichment promoted the restoration of cerebral blood flow in the ipsilesional cortex [6]. Likewise, ( +)Env housing enhanced endothelial cell proliferation and increased the total vessel surface area-induced cerebral angiogenesis that may contribute to the improved neurological outcome of stroke animals after ischemia–reperfusion injury [7].

Quite recently, a combination of plasticity-promoting molecules like EphA4 and enriched environment has been proposed to improve neurobehavioral indices after stroke [8]. In addition, it has been shown that ( +)Env promotes improves cognitive function via synaptic remodeling along with spatial memory and learning ability in the Water Maze test after permanent middle cerebral artery occlusion (pMCAO) [9]. Likewise, housing young mice at two days after stroke in ( +)Env, enhanced tactile-proprioceptive function, possibly via modulation of GABAergic inhibitory interneurons [10]. Similarly, a combination of task-specific reach training and enriched environment, had synergistic effects in single-pellet reaching at 9 weeks post-stroke [11].

At molecular level, increased regenerative capacity induced by enhancing neuronal activity was dependent on Creb-binding protein mediated histone acetylation epigenetic reprogramming in rodent models of spinal cord injury [12]. In another study, environmental enrichment increased the number of synapses in the hippocampal CA1 region along with increased expression levels of growth-associated protein 43, synaptophysin and postsynaptic density protein 95 in the hippocampus [9].

Old age is associated with an enhanced susceptibility to stroke and the aged brain has a limited capacity for spontaneous recovery after cerebral ischemia [13]. However, the majority of experimental studies of stroke have been performed on young animals, which usually recover rapidly during the subacute phase of stroke and therefore may not fully replicate the effects of ischemia on neural tissue in aged subjects. Since stroke afflicts mostly the elderly comorbid patients, it is highly desirable to test the efficacy of stroke therapies in an aged animal model that is clinically most relevant to stroke rehabilitation and cellular studies [14–16]. Here, we investigated how socializing and environmental enrichment contribute to behavioral recuperation after stroke in aged animals.

## Materials and methods

### Animals and experimental design

**Fig. 1:**
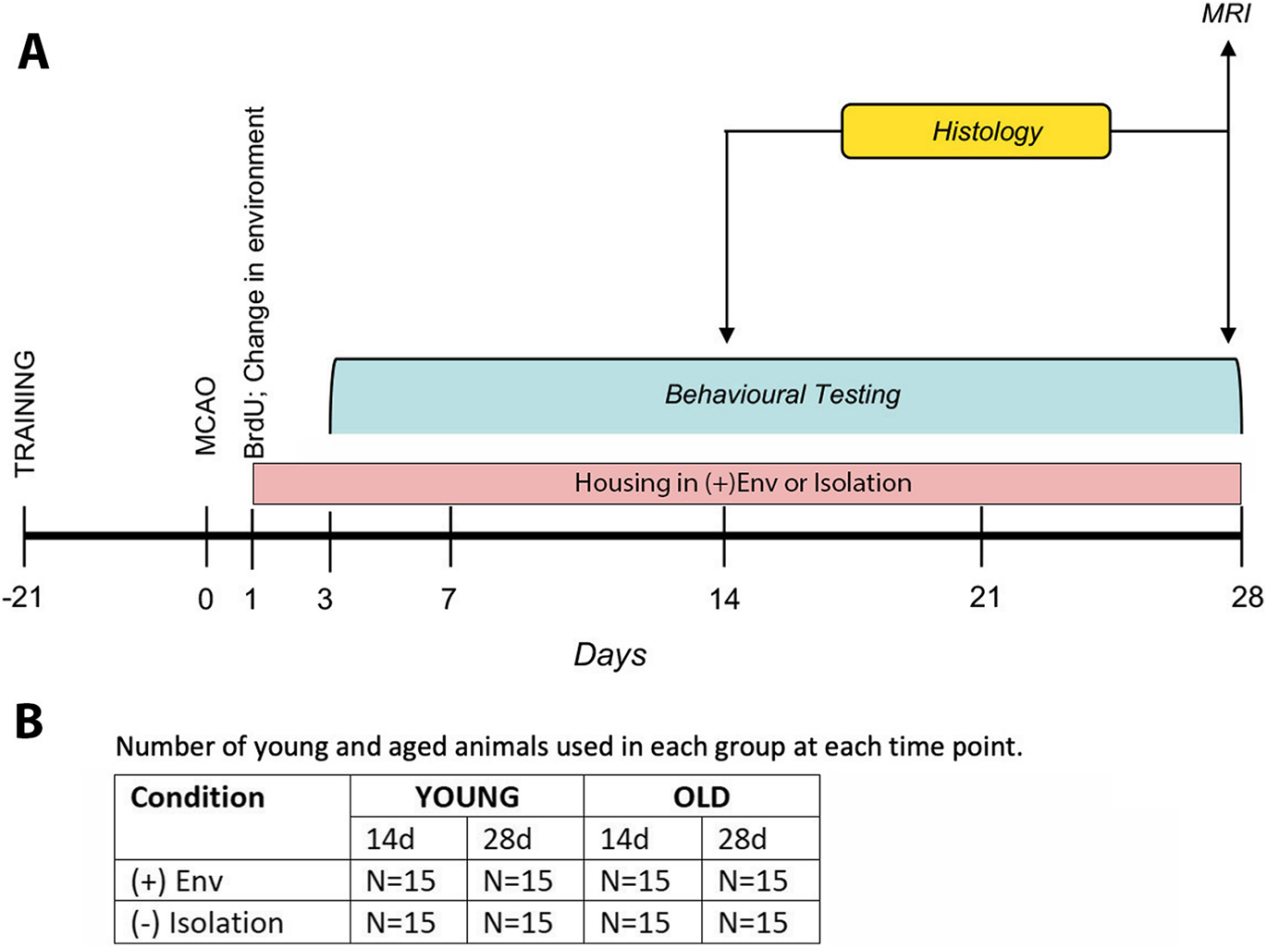
Experimental design (**A**) and the number of young and aged animals used per group and time point (**B**)

Young (4 to 5 months) and aged (19 to 20 moonths) male Sprague–Dawley rats were used. In total, 120 rats bred and reared in our animal facility were used in this study (Fig. 1). Body weights ranged from 310 to 400 g for the young rats and from 550 to 700 g for the aged rats. The rats were kept in groups of three at a temperature of 22 °C, 40–60% humidity, and 07.00–19.00 h light period. The rats had free access to food and water.

### Environment

#### Inhibiting environment isolation

Individual rats were kept in a homemade box (30 × 40 cm) with opaque black side walls that limited the animals' visual field to the room ceiling. Hence, the main source of environmental stimulation for these animals was the behavioral tests themselves, to which all animals in the study were subjected [17].

#### Enriched environment (( +)Env)

Social groups of 10 young and aged rats were placed in a natural habitat-like environment organized in a homemade large cage made of plexiglas and equipped with a running wheel, catwalk, playing toys and hiding tunnels allowing for social grouping and multimodal training including sensorimotor stimulation and motor training.

All experiments were performed in accordance with ARRIVE Guidelines for the Care and Use of Laboratory Animals, and animal procedures were approved by the Griffith University Animal Ethics Committee (Ref. 2017/19).

### Surgery

To minimize the effect of plasma glucose on stroke size, rats that were fasted overnight prior to surgery. The animals were placed in a prone position and the right lateral skull surface surgically exposed. Using a microdrill (Fine Science Tools), a small segment of the skull above the middle cerebral artery was removed 2- to 3-mm rostral to the juncture of the zygomatic arch and the pars squamosa of the temporal bone. The bone was thinned using a low drill speed and superfusion with physiological saline to minimize friction-induced warming. The bone flap was carefully removed with forceps and the underlying dura opened with a fine needle. Then, the right middle cerebral artery (MCAO) was slowly lifted with a tungsten hook attached to a micromanipulator and thermocoagulated as previously described [18]. Both common carotid arteries were then closed for 90 min. Throughout surgery, anesthesia was maintained by spontaneous inhalation of 1–1.5% isoflurane in a mixture of 75% nitrous oxide and 25% oxygen. Body temperature was controlled at 37 °C by a Homeothermic Blanket System (Harvard Apparatus). The local changes in blood flow were monitored using a laser Doppler device (Perimed, Stockholm, Sweden). A decrease in laser Doppler signals to < 20% of control values was considered to be successful MCA occlusion. To minimize post-operative pain and suffering, rats were given 0.05 mg/kg SC buprenorphine (sc). Enriched-environment housing began 1 day after focal ischemia and was maintained for the whole experimental period.

After 90 min, the common carotid arteries were re-opened. Subsequent to survival times of 14 or 28 days, the rats were deeply anesthetized with 2.5% isoflurane in 75% nitrous oxide and 25% oxygen, and perfused with neutral buffered saline followed by buffered 4% freshly depolymerized paraformaldehyde. The brain was removed, post-fixed in 4% buffered paraformaldehyde for 24 h, cryoprotected in 15% glycerol prepared in 10 mmol/l phosphate-buffered saline, flash-frozen in isopentane, and stored at − 70 °C until sectioning.

### Behavioral tests

Testing procedure involved three persons, one person who was in charge of surgery; another one who was in charge of handling the animals according to group assignment and one who tested the animals. To evaluate changes in neurological function associated with ischemia, the rats were subjected to a variety of locomotor, sensory, learning and memory tests before and after surgery [18]. All testing was performed from 9–11 AM by the same investigator. Except inclined plane, 3 weeks before surgery, we gave animals extensive training to reach 100% functionality. Therefore, results obtained before surgery were used to define 100% functionality for each animal on each test, and functional recovery was expressed as percent recovery relative to the pre-surgery baseline.

#### Neurological status

Following surgery, rats were observed for circling behavior if pulled gently by the tail. Rats circling toward the infarcted brain side were ranked as grade 3. Rats that did not circle but fully stretched their forelimbs were graded as 2. Rats that did not circle, but partially stretched their forelimbs were graded as 1. Rats showing no reaction were graded as 0 [18, 19].

#### Rotating pole test

The rotating pole task assesses fine vestibulomotor function in the MCAO model. Each rat was tested for its ability to negotiate a rotating (6 rpm) horizontal rod. The time taken for the rat to traverse the rotating cylinder and join a group of rats visible at the finish line was measured. The score assessment was twofold: (i) time (seconds) required to traverse the rotating cylinder and, (ii) the score as follows: 0, rat falls immediately (onto a soft surface); 1, rat does not walk forward, but stays on the rotating pole; 2, rat walks, but falls before reaching the goal; 3, rat traverses the rod successfully, but the limbs are used asymmetrically; 4, the left forelimb is used less than 50% of the time taken to traverse the rod; 5, the rat successfully traverses the rod, but with a few foot slips; 6, no mistakes, symmetric movements [18].

#### Inclined plane

We tested the ability of each animal to maintain its position at a given angle on an inclined plane [18]. The relative angle at which the rat could no longer maintain its position was taken as a measure of functional impairment. This test was conducted once before surgery and daily thereafter.

#### Spatial learning based on positive reinforcement, working and reference memory

Spatial learning based on positive reinforcement, working, and reference memory was evaluated in the labyrinth food-finding test (T-maze test). Before the test, the animals were deprived of food for 24 h but had unlimited access to water. The rats were trained in the maze test with food placed in the endpoint of a complex route consisting of 3 consecutive T-mazes [18]. The food was the reward for finding the way. The animals always were placed in the same area of the maze (start area). Every 2 days, the rats were fed freely after the test for 30 min. We assigned a score of 0 for successfully finding the baited arm. Because the apparatus was made up of 3 T-mazes, the rats could commit 1, 2, or 3 errors in each trial. The time limit was set to 5 min. Between trials, the maze was cleaned with 1% incidin after the training of each animal to remove olfactory cues.

One day was left between the Neurological status, Rotating pole, Inclined plane and the T-maze test.

### BrdU Labelling

To label newly generated cells, rats were given once daily injections of bromodeoxyuridine (BrdU; 50 mg/ kg body weight, i.p.; Sigma) starting from day 1 and over a period of 14 days.

### Immunohistochemistry

Sections (25 µm thick) were cut on a freezing microtome and processed for immunohistochemistry as previously described [20]. For BrdU detection, free-floating sections were pre-treated with 50% formamide, 0.3 M NaCl, 10 mM sodium citrate at 65 °C for 2 h, incubated in 2 M HCl at 40 °C for 1 h, and rinsed in 0.1 M borate buffer (pH 8.5) at room temperature for 10 min. Sections were incubated with the mouse monoclonal anti-BrdU antibody (1:300, Roche, Mannheim, Germany) at 4 °C for 24 h.

#### Phenotyping of ED1/BrdU cells

Sections were incubated with the rat anti-BrdU (1:2000, Serotec, UK) at 4 °C for 24 h. BrdU-positive nuclei were visualized using goat anti-rat-Cy3. After a short fixation step of 10 min, sections were rinsed with PBS and incubated with mouse anti-CD68 (ED1) (1:1000, abcam, UK) followed by goat anti-rat-Cy2.

#### Phenotyping of nestin/BrdU cells

Sections were incubated with the rat anti-BrdU (1:2000, Serotec, UK) at 4 °C for 24 h. BrdU-positive nuclei were visualized using goat anti-rat-Cy2. After a short fixation step of 10 min, sections were rinsed with PBS and incubated with mouse anti-nestin (clone Rat-401, 1:3000, abcam, UK) followed by goat anti-mouse-Cy3.

#### Phenotyping of DCX/BrdU cells

Sections were incubated with the rat anti-BrdU (1:2000, Serotec, UK) at 4 °C for 24 h. BrdU-positive nuclei were visualized using goat anti-rat-Cy3. After a short fixation step of 10 min, sections were rinsed with PBS and incubated with mouse guinea pig-DCX (1:3000, Millipore, Germany) followed by goat anti-rat-Cy2.

#### Phenotyping of DCX/BrdU cells

Sections were incubated with the rat anti-BrdU (1:2000, Serotec, UK) at 4 °C for 24 h. BrdU-positive nuclei were visualized using goat anti-rat-Cy3. After a short fixation step of 10 min, sections were rinsed with PBS and incubated with rabbit anti-tubulin beta III (1:5000; abcam, UK) followed by goat anti-rat-Cy2.

### Counting of co-localized cells

ED1/BrdU-, DCX/BrdU-, nestin/BrdU-, and tubulin beta III/BrdU-positive cells were analyzed in every 10th section in the region adjacent to the scar-confined area corresponding to the formerly infarct core and subventricular zone as previously described [19]. To this end, a sequence of confocal counting images of 161 × 242 × 25 µm, spaced 0.4–0.6 µm apart across a 25-µm-thick section and covering 30% of the infarcted area, was taken for fluorescently labeled cells [21]. The relative mean number of double labelled cells was then calculated by multiplying the number of cells per section times 3.3 (the counting boxes that were quantitated covered one third of the area of each section) times the section interval of 10.

### Determination of Infarct Volume by MRI

Magnetic resonance imaging (MRI) was used to visualize the infarct volume for all groups at day 28 after stroke. MRI measurements were performed on a 7-T Bruker ClinScan magnet with a 20 cm inner bore, capable of 290 mT/m in 250 µs (Bruker BioSpin MRI, Ettlingen, Germany) [22]. Images were received by a 2 × 2 phased array RF coil, designed specifically for rat brain studies that was placed directly on the skull. The animals were anesthetized during imaging to minimize discomfort. Respiratory rate was monitored, and isoflurane concentrations were varied between 1.5 and 2.0% to keep the respiratory rate between 35 and 45/min. After positioning the animal’s head, T2-weighted MRI was recorded were performed with a multislice spin-echo sequence with 25 slices of 0.7 mm thickness and a matrix size 640 × 640 pixels, field-of-view of 32 × 32 mm, a repetition time (TR) of 4330 ms, and an echo time (TE) of 45 ms [18].

### Lesion measurement using MIPAV software

T2WI lesion volumes were determined using the image processing software Medical Image Processing, Analysis and Visualization (MIPAV, version 3.0, National Institutes of Health, Bethesda, MD, USA). After optimal adjustment of contrast, the edge of the lesion was traced manually on each of the 25 coronal slices, which completely covered the MCA territory in all animals. The areas of hyperintensity were then summed and multiplied by the slice thickness to calculate lesion volumes as previously described [22].

#### Microscopy

For light microscopy, a Nikon Eclipse (Nikon, Duesseldorf, Germany) was used. Confocal microscopy images were acquired using a Zeiss LSM710 laser-scanning confocal system with spectral detection capabilities, and Zen 2010 software version 6.0 (Carl Zeiss Microscopy GmbH, Jena, Germany) was used for image acquisition and analysis. Excitation light was provided by 488, 543, and 634 nm laser lines; fluorescence emission was detected at 500–530 nm for Cy2 (green) and 550–600 nm for Cy3 (red) in separate tracks, using a confocal aperture of 1 Airy unit. Some of the images were acquired as z-stacks and 3D reconstruction was performed.

#### Statistical analysis

The effect of age, environment, time, as well as their interactions on behavioral recovery for young and old animals was analyzed using three-way ANOVA. Between-group comparisons were done by two-way ANOVA followed by Sidak’s multiple comparisons test. For the analysis of non-parametric data (neurological status) we used the Friedman test followed by Dunn’s multiple comparisons test. For the analysis of histological data, we used two-way ANOVA followed by Tukey’s multiple comparisons test for assessing the effect of treatment on the number of co-localized DCX/BrdU, TubIII/BrdU, ED1/BrdU using GraphPad software. The level of significance was set at *P* = 0.05. The relationship between metric data and histological data (such as cell count or infarct volume) was assessed using Pearson’s product moment correlation (Pearson’s *r*). The relationships between data from several tests (neurological status, labyrinth, inclined plane, rotating pole) and histological parameters (such as cell counts) were evaluated by rank correlations whereby rank 1 was assigned to animals that achieved the best performance, or had the lowest cell number as previously described [17]. For such calculations we used Kendall’s tau-b correlation test.

## Results

### General observations

In the first 24 h after surgery, the animals were somewhat listless, probably in part due to the aftereffects of the anesthesia. This condition improved to some extent in the ensuing days, although the animals tended to remain sensitive to external stimulation and noises.

To mitigate problems of feeding in aged animals during the first 3 days post-stroke, we fed them with moistened, soft pellets. Nevertheless, aged animals do lose some weight in the first week following stroke. The mortality rate was higher for aged rats (23%) than for younger rats (10%).

The time to acquire 100% functionality was different depending on the age. While the young animals fulfilled the training requirements in about 2 weeks, the old rats needed about 3 weeks to achieve 100% functionality. Following infarction, all rats had diminished performance on the first post-surgical day, part of which was attributable to the surgery itself. In addition, the aged rats were more severely impaired by stroke and showed delayed functional recovery compared with young rats.

### Behavioral analysis

#### Neurological status

**Fig. 2:**
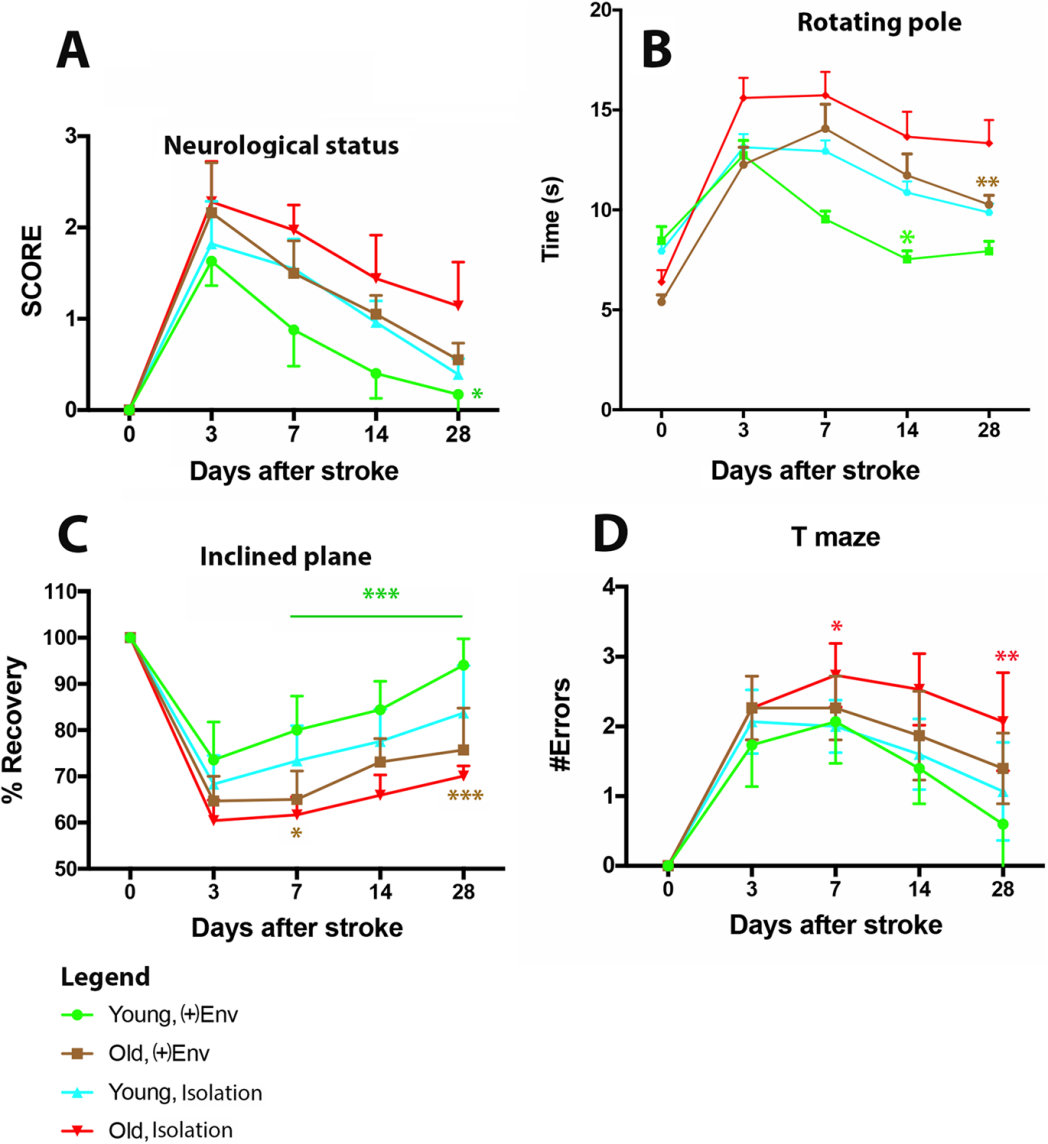
Effect of age and environment on behavioral recovery after stroke. Note that the old rats kept in the ( +)Env reached performance levels on neurological status similar to those achieved by the young animals kept in isolation (**A**; brown line). On rotating pole, the old rats kept in isolation performed worst. However, the enriched environment had a significant beneficial effect and allowed aged rats to recover to the levels attained by young rats kept in isolation (**B**; green line vs. red line). On the inclined plane, the ( +)Env had a beneficial effect on recovery in old rats (**C**, brown vs red line). Recovery of spatial memory in old rats kept in a ( +)Env was significantly better than that in old rats kept in isolation (**D**; brown vs. red line). The effect of ( +)Env had, nevertheless, no significant effect on recovery in young rats in this test. Data are shown as mean ± SD. *P = 0.01; **P = 0.001; ***P = 0.0001

In the first 3 days, their performance declined in all groups and animals began to recover by day 3 post-stroke. However, even by the 28th day, the young animals kept in isolation did not achieve their baseline performance (Fig. 2A, blue line). The young rats kept in ( +)Env performed significantly better (*P* = 0.0197) than old rats kept in isolation. Nevertheless, the old rats kept in isolation recovered up to 50% of their baseline performance (Fig. 2A, red line). The ( +)Env had a beneficial effect on the NS recovery in old rats which reached performance levels similar to those achieved by the young animals kept in isolation (Fig. 2A, brown line).

#### Rotating pole

Both young and old post-stroke animals groups benefited from the ( +)Env starting with days 3–7 after stroke. By 3-way ANOVA, time, environment, and age were highly significant (*P* < 0.0001) for the recovery of performance on the inclined plane. Likewise, time x age was also significant (*P* = 0.0012) was beneficial in recovery of function in this test. Time × Env was close to significance (*P* = 0.073). However, neither Env x age (*P* = 0.656) nor time x Env age (*P* = 0.681) were significant (Table 1 s).

**Young rats.** By 2-way ANOVA, both ( +)Env [*F*(1, 14) = 36.67; *P* < 0.0001] and time [*F*(4, 56) = 35.10; *P* < 0.0001] had a significantly positive effect on the time course of recovery on the rotating pole starting with day 3 after stroke (*P* = 0.0021) but become less significant (*P* = 0.0134) at the end of the testing period. Young rats kept in an ( +)Env were on the average, faster than aged rats kept in an enriched environment (Fig. 2B, green vs brown line).

**Old rats.** Aged rats kept in isolation performed worst. However, both time [*F*(4,56) = 144.2; *P* < 0.0001] and enriched environment had a highly significant [*F*(1,14) = 23.01; *P* = 0.0003] beneficial effect on recovery starting with day 7 after stroke (*P* = 0.0161) and allowed aged rats to recover to the levels attained by young rats kept in isolation (Fig. 2B; brown line vs. blue line) (*P* < 0.0001).

Of note, the effect of ( +)Env was significantly higher (*P* < 0.0001) in young animals as compared to old rats (Fig. 2B, green vs brown line).

#### Inclined plane

By 3-way ANOVA, age, environment, time, and time × age were highly significant for the recovery of performance on the inclined plane. Likewise, time x Env was also significant (*P* = 0.0065) was beneficial in recovery of function in this test. However, neither Env × age (*P* = 0.1906) nor time × Env × age (*P* = 0.1377) were significant (Table 1 s).

##### Young rats

By 2-way ANOVA, both time [*F*(4,56) = 220; *P* < 0.0001] and enriched environment had a highly significant [*F*(1,14) = 9.644; *P* = 0.0077] beneficial effect on recovery. Functional recovery in young rats kept in an ( +)Env started at day 3 (*P* = 0.0012) as compared to the animals kept in isolation and reached the preoperative baseline level by the end of the testing period (*P* ≤ 0.0001) (Fig. 2C, green vs blue line).

##### Old rats

By 2-way ANOVA, both time [*F*(4,56) = 491.9; *P* < 0.0001] and enriched environment had a highly significant [*F*(1,14) = 12.3; *P* = 0.0035] beneficial effect on recovery. The ( +)Env had a delayed positive effect on recovery in old rats who started to recover later, by day 14 (*P* < 0.0001) (Fig. 2C, brown vs red line). Of note, the effect of ( +)Env was significantly greater in young animals as compared to old rats (Fig. 2C, green vs brown line). Of note, the effect of ( +)Env was significantly greater (*P* < 0.0001) in young animals as compared to old rats (Fig. 2C, green vs brown line).

#### Labyrinth (T-maze)

By 3-way ANOVA, time, environment, age, and time × age were highly significant (*P* ≤ 0.0001) for the recovery of spatial learning based on positive reinforcement, working and reference memory. Likewise, time × Env was beneficial (*P* = 0.0132) in recovery of function in this test. However, neither Env × age (*P* = 0.124) nor time × Env × age (*P* = 0.0958) were significant (Table 1 s). Recovery of spatial memory was clearly impaired after stroke in both age groups and performance in this test deteriorated progressively. Animals began recover by day 7 and young animals kept in ( +)Env were almost error-free by day 28 post-stroke, as compared to day 7 post-stroke when the number of errors was highest (Fig. 2D).

##### Young rats

By 2-way ANOVA, both time [*F*(4,56) = 122.2; *P* < 0.0001] and enriched environment had a significantly [*F*(1,14) = 5.565; *P* = 0.0344] beneficial effect on recovery. Functional recovery in young rats kept in an ( +)Env started at day 14 as compared to the animals kept in isolation and reached the preoperative baseline level by the end of the testing period (Fig. 2D, green vs blue line). The ( +)Env had, nevertheless, no significant effect on recovery in this test.

##### Old rats

By 2-way ANOVA, both time [*F*(4,56) = 136.8; *P* < 0.0001] and enriched environment had a highly significant [*F*(1,14) = 36.98; *P* < 0.0001] beneficial effect on recovery. The ( +)Env had a delayed positive effect on recovery in old rats who started to recover later, by day 14 (*P* = 0.0014) (Fig. 2D, brown vs red line). Of note, the effect of ( +)Env was significantly greater (*P* = 0.0019) in young animals as compared to old rats (Fig. 2D, green vs brown line).

#### Enriched environment does not significantly reduce infarct volume

**Fig. 3:**
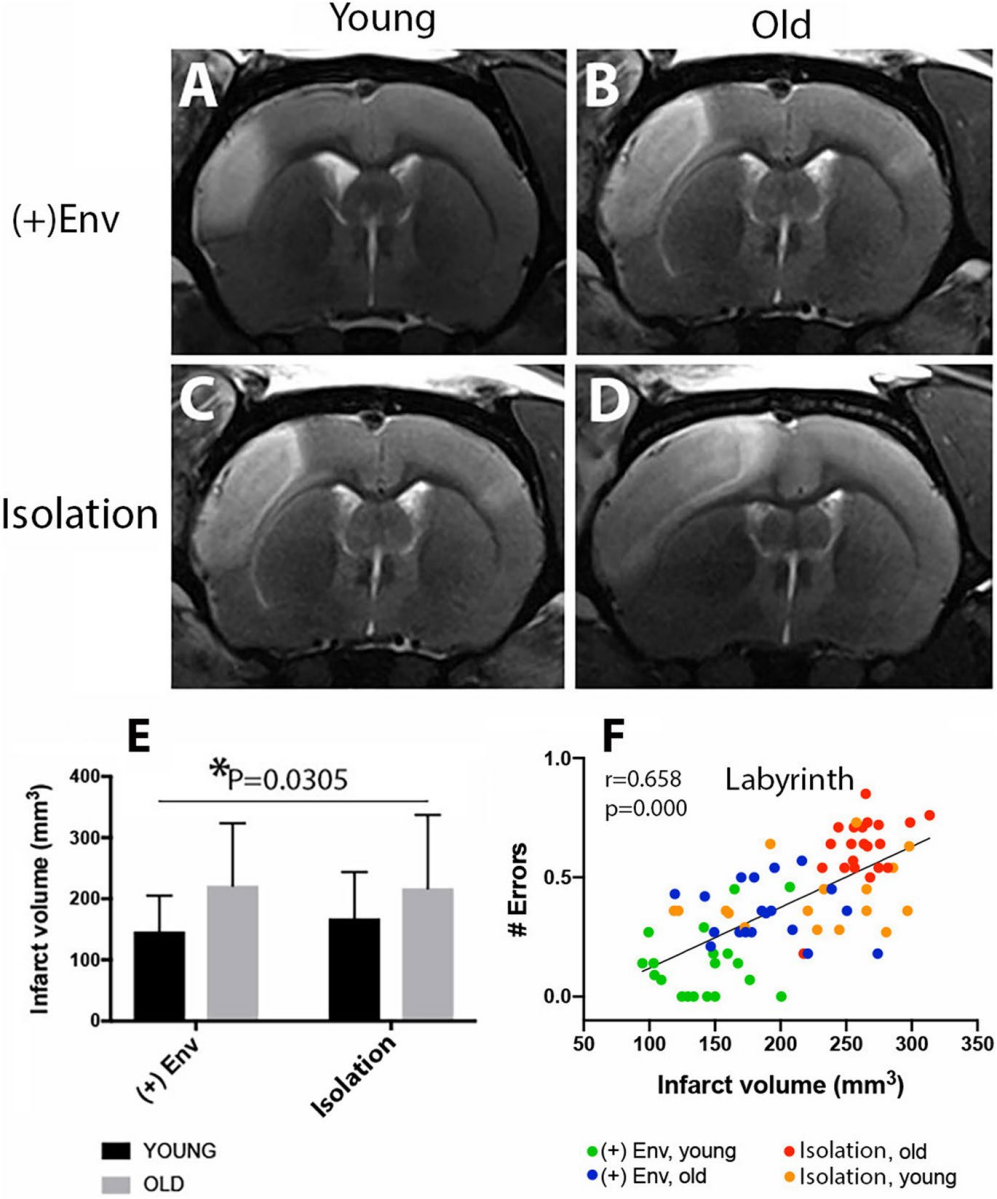
The effect of environment on infarct size in young and aged animals. Note that by two-way ANOVA, old animals had significantly larger infarcts as compared to young animals. However, environment did not exert a significant effect on the infarct volume in either group (**A**, **B** vs **C**, **D**). A smaller scar volume positively correlates with better recovery of spatial learning based on positive reinforcement, working and reference memory of young and to a lesser extent in old animals kept in ( +)Env (**F**). Data are shown as mean ± SD

Representative MRI data for rats closest to the mean for each group are shown in Fig. 3. By two-way ANOVA, old animals had significantly larger infarcts as compared to young animals [*F*(1,40) = 5.028; *P* = 0.0305; Fig. 3E]. However, environment did not exert a significant effect on the infarct volume in either group (Fig. 3A, B vs C, D). Correlation analysis was used to assess potential relationships between infarct volume and recovery of performance in the labyrinth test. We found that a smaller infarct volume positively correlates with better recovery of spatial learning based on positive reinforcement, working and reference memory of young and, to a lesser extent, old animals kept in ( +)Env (Fig. 3F).

#### Isolation increased the number of inflammatory cells in the old brains

**Fig. 4:**
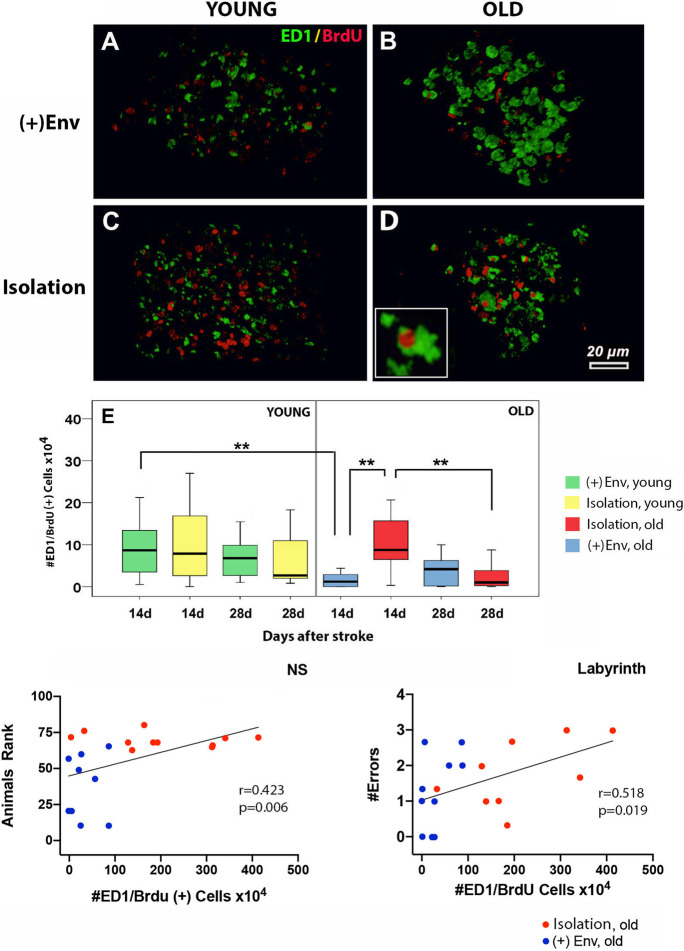
The effect of environment on the number of proliferating ED1/BrdU cells in the peri-infarcted area of young and aged rats and performance in neurobehavioral tests. At day 14 after stroke, the number of ED1/BrdU cells in the peri-infarcted area of young rats was not significantly changed by the ( +)Env (**A** vs **C**; **E**). However, isolation increased the number of inflammatory cells in old brains (**B** vs **D**; **E**). By day 28, the number of ED1/BrdU cells in the peri-infarcted area of old rats was no longer significant (**E**). Correlation analysis revealed that a lower number of ED1/BrdU cells in the peri-infarcted area of old animals kept in ( +)Env, correlated positively with performance on neurological status (**F**) and spatial memory (F, G). Data are shown as mean ± SD

Anti CD68 antibody (ED1) is routinely used as a histochemical marker of inflammation associated with the monocytes/macrophages. At day 14 after stroke, the number of ED1/BrdU cells in the peri-infarcted area of young rats was not significantly changed by the ( +)Env (Fig. 4A vs C; Fig. 4E). However, at day 14, the number of ED1/BrdU cells in the peri-infarcted area of old rats was significantly higher in the lesioned area of rats kept in isolation [*F*(1,19) = 14.14; **P < 0.004] (Fig. 4B vs D; Fig. 4E). By day 28, the number of ED1/BrdU cells in the peri-infarcted area of old rats was no longer significant between isolation and ( +)Env (Fig. 4E). Quite interestingly, the number of ED1/BrdU cells in the peri-infarcted area of young rats kept in ( +)Env was significantly higher than that of old rats kept in ( +)Env [*F*(1,19) = 10.40; **P < 0.007] (Fig. 4B vs D; Fig. 4E). Correlation analysis was used to assess the relationship between the number of ED1/BrdU cells in the peri-infarcted area and recovery of performance in behavioral tests. We found that a lower number of ED1/BrdU cells in the peri-infarcted area of old animals kept in ( +)Env, correlated positively with performance on neurological status (Fig. 4F) and spatial memory (Fig. 4F, G).

#### Environmental enrichment and young age increased the number of neuroepithelial cells in the damaged brain area

**Fig. 5:**
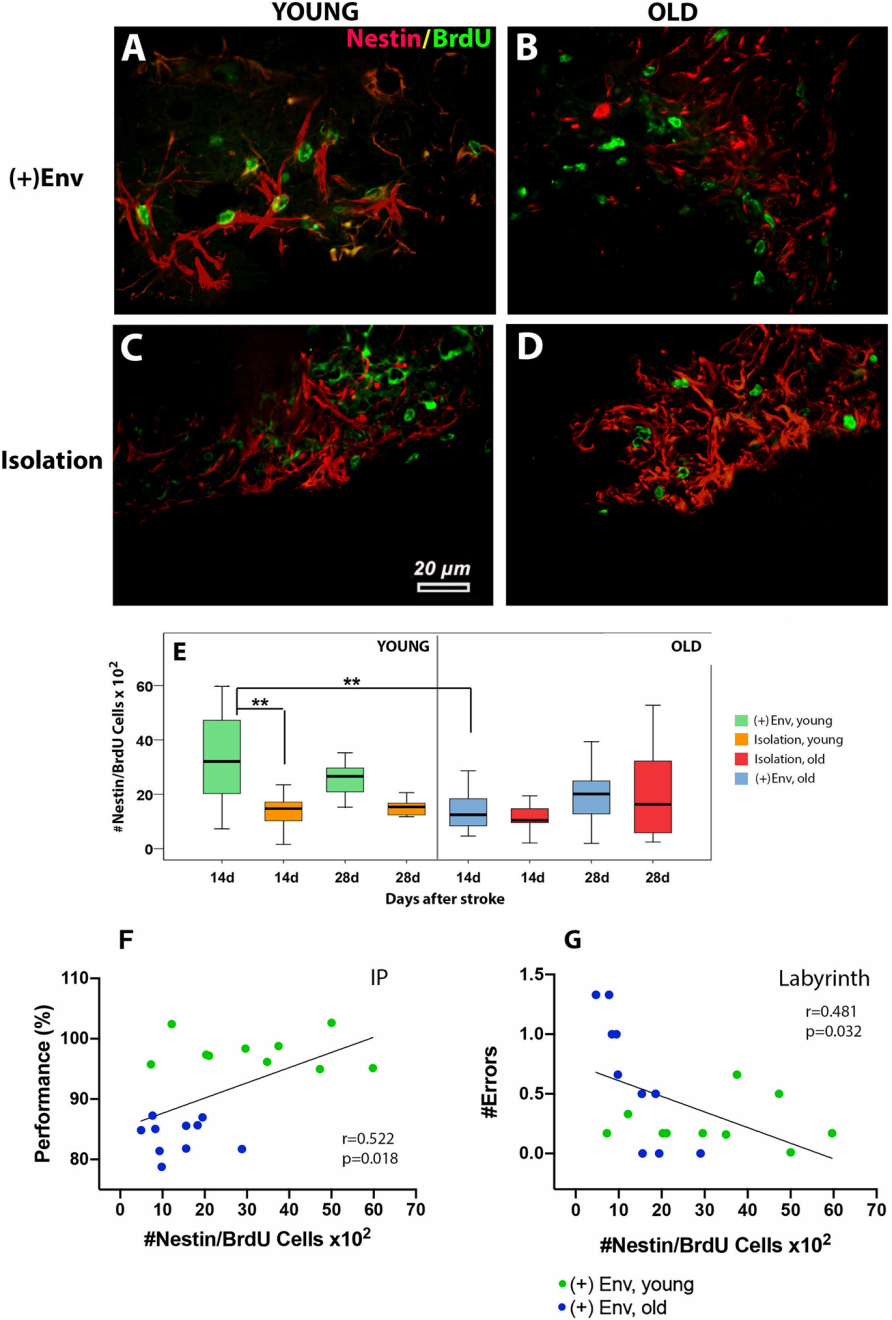
The effect of environment on the number of proliferating neuroepithelial cells in the damaged brain area and performance in neurobehavioral tests. ( +)Env exerted a positive effect on the number of Nestin/BrdU cells in the peri-infarcted area of young rats as compared to young rats kept in isolation (**A** vs **C**; **E**). However, by day 28, neither age nor the environment exerted a significant effect on the number of nestin/BrdU cells (**A**, **B** vs **C**, **D**; **E**). Correlation analysis between the number of nestin/BrdU cells in the lesioned area and performance indicated a positive correlation for the inclined plane (**F**) and a negative correlation in the labyrinth test (**G**). Data are shown as mean ± SD

Nestin is a marker of proliferating pericytes and astrocytes that detach from damaged blood vessels after stroke [23]. At day 14 after stroke, the ( +)Env exerted a positive effect on the number of nestin/BrdU cells in the peri-infarcted area of young rats as compared to young rats kept in isolation [*F*(1,19) = 10.34; **P = 0.005] (Fig. 5A vs C; Fig. 5E). At this time point, age exerted also a significant effect on the number of nestin/BrdU cells in the peri-infarcted area [*F*(1,19) = 9.58; **P = 0.008]. However, by day 28, neither age nor the environment exerted a significant effect on the number of nestin/BrdU cells (Fig. 5A, B vs C, D; Fig. 5E). Correlation analysis between the number of nestin/BrdU cells in the lesioned area and performance indicated a positive correlation for the inclined plane (Fig. 5F) and a negative correlation in the labyrinth test (Fig. 5G).

#### Environmental enrichment and an increased number of DCX/BrdU cells exerted a significant effect on performance

**Fig. 6:**
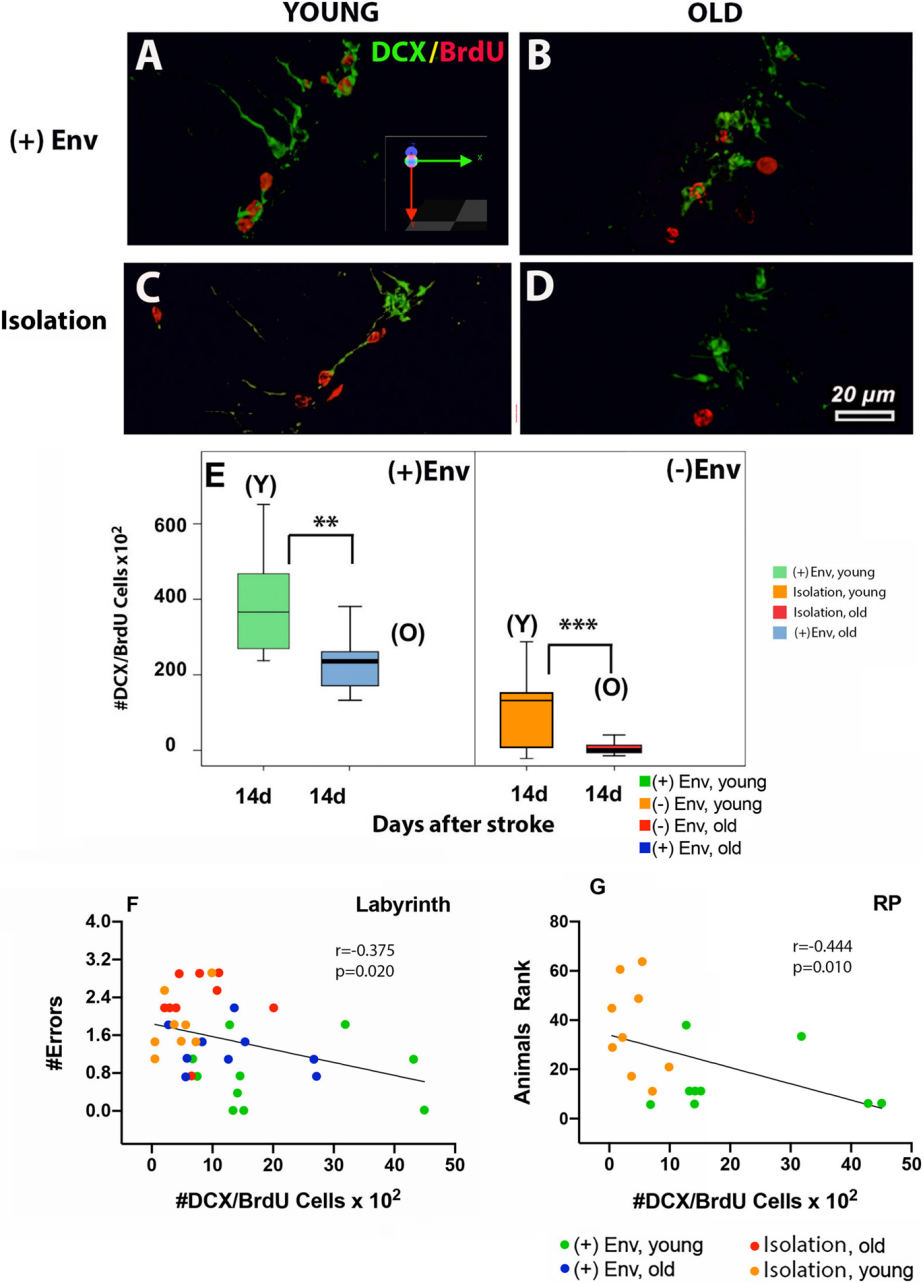
The effect of environment on the number of proliferating neuronal precursor cells in the damaged brain area and performance in neurobehavioral tests. At day 14 after stroke, the number of DCX/BrdU cells in the peri-infarcted area of both young and old rats was significantly higher in the brains of rats kept in the ( +)Env (**A**, **B** vs **C**, **D**; **E**). Correlation analysis indicated that environmental enrichment and an increased number of DCX/BrdU cells exerted a significant effect on performance for working memory (**F**) and on the rotating pole (**G**). Data are shown as mean ± SD

Doublecortin (DCX), a microtubule-associated phosphoprotein, has become a widely accepted marker for newly born neurons. At day 14 after stroke, the number of DCX/BrdU cells in the peri-infarcted area of both young and old rats was significantly higher in the brains of rats kept in the ( +)Env [*F*(1,19) = 11.38; **P = 0.005] (Fig. 6A, B vs C,D; Fig. 6E). Young age kept in either condition, exerted a significant effect on the total number of DCX/BrdU cells in the subventricular zone, ipsilateral to the lesion as compared to old rats (Fig. 6E). However, there was no significant change in the number of DCX/BrdU cells from day14 to day28 (not shown). Correlation analysis indicated a positive correlation between working memory (Fig. 6F) and performance on the rotating pole (Fig. 6G) between the number of DCX/BrdU cells in the subventricular zone, ipsilateral to the lesion. Specifically, both young and old rats kept in isolation made consistently more errors in the labyrinth test as compared to animals kept in ( +)Env (Fig. 6F). Likewise, environmental enrichment and an increased number of DCX/BrdU cells exerted a significant effect on performance on the rotating pole (Fig. 6G).

#### Young age and enriched environment exerted a significant effect on beta III tubulin expression in the perilesional area

**Fig. 7:**
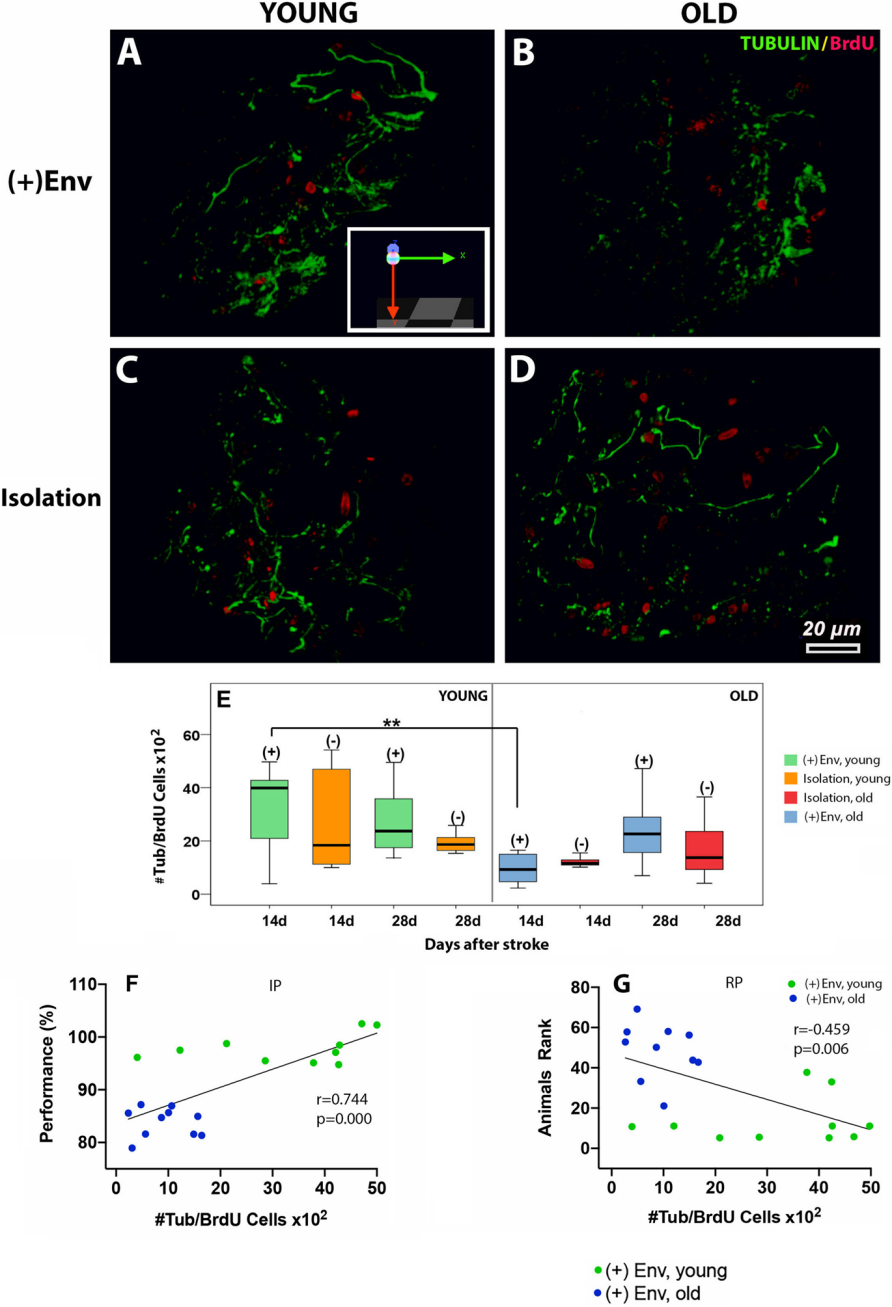
The effect of environment on the number of beta III tubulin expression in the perilesional area and performance in neurobehavioral tests. At day14 post-stroke, young age and ( +)Env exerted a significant effect on beta III tubulin expression in the perilesional area (**A**, **C** vs **B**, **D**; **E**). Correlation analysis indicated a positive effect of young age and ( +)Env on the correlation between the number of beta III tubulin/BrdU cells and performance on the inclined plane (**F**) and rotating pole (**G**). Data are shown as mean ± SD

Beta III tubulin expression correlates with the earliest phases of neuronal differentiation. We found that at day 14 post-stroke, young age and ( +)Env exerted a significant effect on beta III tubulin expression in the perilesional area. Thus, the total number of beta III tubulin/BrdU cells in the peri-infarcted area of young kept in the ( +)Env was significantly higher [*F*(1,19) = 20.24; **P = 0.001] as compared to that of old rats kept in the ( +)Env (Fig. 7A, C vs B, D; Fig. 7E). At day 28, however, neither age nor the environment exerted a significant effect on the number of beta III tubulin/BrdU cells. Correlation analysis indicated a positive effect of young age and + (Env) on the correlation between the number of beta III tubulin/BrdU cells and performance on the inclined plane (Fig. 7F) and rotating pole (Fig. 7G).

## Discussion

There is evidence suggesting that patients in rehabilitation wards, apart from their scheduled therapy sessions, spend most of their time physically inactive and relatively isolated [24]. Previous studies after stroke suggest that engagement in physical, cognitive, and social activities likely reduce a patient’s boredom and improve recovery [25–28]. Given that stroke recovery depends on neuroplasticity, environmental enrichment is viable option for stimulating neural recovery through physical, cognitive and social activities [29]. However, the cytological and molecular mechanisms underlying an improved recovery in a stimulating environment are not precisely known.

In animal models, socialization and physical enrichment is an important component of ( +)Env and positively contributes to improved behavioral recuperation after stroke in young animals [30]. However, since stroke is primarily an affliction of older people, it is important to evaluate the effects of environmental factors on functional restoration in an aged animal model. Here we show that early exposure of post-stroke old rats to a stimulating social-, motor and sensory environment, is effective in improving behavioral recovery (working memory and rotating pole) after stroke in old rats and was associated with reduced neuroinflammation and increased number of neuronal precursors expressing doublecortin. Old age, however, exerted a small but significant effect on lesion size, which was independent of the environment.

By two-way ANOVA, the enriched environment and young age significantly improved functional recovery for neurological status, rotating pole and to a lesser extent, the inclined plane whereby the recovery was time-dependent, but had no significant effect on recovery of memory and spatial learning in the labyrinth test. In aged rats, environmental enrichment significantly improved functional recovery in all tests. However, for the inclined plane and exploratory behavior, recovery was time-dependent.

In our study, enriched environment did not significantly reduce infarct volume in either group. This is in good agreement with previous studies that showed in adult rats it has been shown that environmental enrichment restores function but does not attenuate the infarct volume [31]. However, old rats had larger infarcts most likely due to an increased Inflammatory response. Indeed, a recent study showed larger infarcts in old mice were associated with more severe neuronal injury after ischemic stroke [32].

In young animals, inflammasome-mediated inflammation aggravates post-stroke cognitive impairment possibly by pyroptosis-induced neuronal death [14]. Aged rat brains mount a massive neuroinflammatory response during the acute phase of stroke [33], and neuronal damage is more severe in 12-month-old mice after ischemic stroke than in 2-month-old mice, which could be attributed to the increased number of inflammatory cells and levels of pro-inflammatory cytokines [32]. In the current study, isolation/impoverishment increased the number of inflammatory cells in the lesioned area of old but not young rats. Of interest, old animals kept in ( +)Env performed better than old animals kept in isolation suggesting that neuroinflammation in the aged brains is detrimental for behavioral recovery. Indeed, it has been reported that ( +)Env can improve post-stroke cognitive decline by inhibiting neuroinflammation and oxidative stress [34].

We have previously reported that the capillaries of the corpus callosum of the aged brain are the major source of proliferating, neuroepithelial, nestin-positive cells shortly after cerebral ischemia [23]. Since most of the proliferating nestin-positive cells will contribute to the formation of the axonal growth-inhibitory glial scar, it is not surprising that animals kept in isolation performed worst.

There are also reports that delayed exposure to ( +)Env may have a similar beneficial effect on spatial learning and enhanced memory after brain injury possibly via increased hippocampal and STAT3-HIF-1α-VEGF-mediated neurogenesis in the subventricular zone in young animals [35–37]. Indeed, we found that both the environmental condition and young age exerted a positive effect on motor and spatial memory. Likewise, ( +)Env with a complex combination of sensorimotor, cognitive and social stimulations, enhanced neurovascular reorganization and improved poststroke cognitive function in young rats [34].

Beta-III tubulin is a marker of immature neurons [38]. In our experimental setup, both the ( +)Env and young age exerted a positive effect on grasping and motor coordination on the inclined plane and vestibulomotor function on the rotating pole suggesting that an increased survival rate for lesion-generated immature neurons in the brains of young animals. On these two motor tests, the ( +)Env did not improve performance in old rats. Indeed, there are reports on the occurrence of neurogenesis in the cerebral cortex following stroke [39–41] even in the post-stroke human cortex [42]. The origin of newly born neurons seems to be the subventricular zone [43–45]. Indeed, depleted DCX-expressing and BrdU-labeled cells from the rostral subventricular zone and dentate gyrus, and abolished neurogenesis and associated migration and exacerbated post-ischemic sensorimotor behavioral deficits measured by rotarod, limb placing, and elevated body swing tests [46].

Although stroke patients highly value physical activity and believe that physical activity levels are highly related to enhanced recovery, a large body of evidence has demonstrated that stroke patients in hospital spend most of the time resting in bed and show low levels of social and cognitive activity [47]. Therefore, the benefits observed in the preclinical environment have encouraged clinicians to translate the potential of ( +)Env as an adjunctive therapeutic to human clinical practice. Therefore, it is advisable to explore stroke survivors’ experience of implementation of exposure to an ( +)Env within a typical stroke rehabilitation setting, in order to identify facilitators and barriers to participation. Thus, semi-structured interviews with 10 old stroke survivors placed in a ( +)Env for a 2-week period within a stroke rehabilitation ward provided preliminary support in terms of increased motor, cognitive and sensory stimulation as well as increased social interaction, for the implementation of ( +)Env from a patient perspective [48–50]. While research on stroke patients cannot always probe the same biological mechanisms available in animal models, patient studies can use data collected preclinically to identify useful biomarkers which could direct an accurate stratification of patients in clinical trials [51, 52].

## Conclusions

We provide evidence that a stimulating social, motor and sensory environment had a significant positive effect on recovery on the rotating pole, the inclined plane and labyrinth test in old animals. Old age, however, exerted a small but significant effect on lesion size, which was independent of the environment. Further, a smaller infarct volume positively correlated with better recovery of spatial learning based on positive reinforcement, working and reference memory of young, and to a lesser extent, old animals kept in ( +)Env. Enriched environment and young age had a significant positive effect on behavioral recovery on rotating pole, inclined plane, starting early after stroke. Histologically, isolation was associated with an increased the number of proliferating inflammatory cells expressing ED1 cells in the peri-infarcted area of old but not young rats. Consequently, old animals kept in ( +)Env had a lower number of number of ED1/BrdU cells in the peri-infarcted positively which correlated with performance recovery on neurological status and spatial memory. Further, environmental enrichment and young age were associated with an increased the number of neuroepithelial cells expressing nestin/BrdU as well as beta III tubulin cells in the damaged brain area which correlated with an increased performance on the inclined plane and rotating pole. Finally, environmental enrichment and an increased number of DCX/BrdU cells, exerted a significant effect on performance for working memory and performance on the rotating pole and labyrinth in both age groups.

## Supplementary Information

Below is the link to the electronic supplementary material.

Supplementary file1 (JPG 40 KB)

Supplementary file2 (PDF 42 KB)

## Declarations

### Data availability

Data will be made available to qualified researchers upon request.
