# Supplementary figures and images for: Effect of environmental enrichment and isolation on behavioral and histological indices following focal ischemia in old rats
Source: GeroScience. 2021 Aug 12;44(1):211–28. doi: 10.1007/s11357-021-00432-z (PMC8811116; doi:10.1007/s11357-021-00432-z)

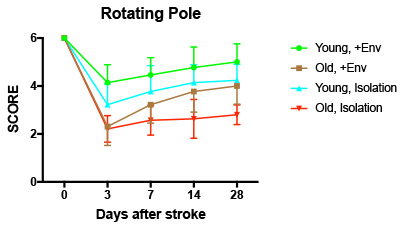

Supplement: Supplementary file 1 — Supplementary file1 (JPG 40 KB) [file 11357_2021_432_MOESM1_ESM.jpg]
