# Supplementary material for: Effect of environmental enrichment and isolation on behavioral and histological indices following focal ischemia in old rats
Source: GeroScience. 2021 Aug 12;44(1):211–28. doi: 10.1007/s11357-021-00432-z (PMC8811116; doi:10.1007/s11357-021-00432-z)
Supplement: Supplementary file 2 — Supplementary file2 (PDF 42 KB) [file 11357_2021_432_MOESM2_ESM.pdf]

Table 1s. Effect of age, environmental enrichment and time on behavioral recovery by three-way ANOVA.

**Test**	**Time**	**Env**	**Age**	**Time** x **Env**	**Time** x **Age**	**Env** x **Age**	**Time** x **Env** x **Age**
**Inclined plane**	F (4, 280) = 212.9 P<0.0001	F (1, 280) = 26.31 P<0.0001	F (1, 280) = 80.03 P<0.0001	F (4, 280) = 3.649 P=0.0065	F (4, 280) = 8.003 P<0.0001	F (1, 280) = 1.772 P=0.1906	F (4, 280) = 1.377 P=0.1377
**Labyrinth**	F (4, 280) = 212.2 P<0.0001	F (1, 280) = 23.68 P<0.0001	F (1, 280) = 75.06 P<0.0001	F (4, 280) = 3.28 P=0.0132	F (4, 280) = 7.415 P<0.0001	F (1, 280) = 2.38 P=0.124	F (4, 280) = 1,7993 P=0.0958
**Rotating pole**	F (4, 180) = 86.02 P<0.0001	F (1, 180) = 27.6 P<0.0001	F (1, 180) = 74.61 P<0.0001	F (4, 180) = 2.169 P=0.073	F (4, 180) = 4.73 P=0.0012	F (1, 180) = 0.1982 P=0.656	F (4, 180) = 0.5749 P=0.681
